# Egg consumption and risk of cardiovascular disease: a PERSIAN cohort-based study

**DOI:** 10.1186/s12872-023-03621-0

**Published:** 2023-11-30

**Authors:** Golsa khalatbari Mohseni, Saeideh Mohammadi, Zohreh Aghakhaninejad, Shirin Tajadod, Khadijeh Abbasi, Seyed Ali Askarpour, Zahra Salimi, Hanieh Shafaei Kachaei, Asma Rajabi Harsini, Farkhondeh Alami, Seyedeh Elaheh Bagheri, Seyed Alireza Mosavi Jarrahi, Ali Gohari, Sara Khoshdooz, Saeid Doaei, Akram Kooshki, Maryam Gholamalizadeh

**Affiliations:** 1 Nutrition and Metabolic Diseases Research Center, Ahvaz Jundishapur University of Medical Sciences, Ahvaz, Iran; 2 Department of Nutrition, Zanjan University of Medical Sciences, Zanjan, Iran; 3 Department of Nutrition and Biochemistry, School of Health, Kerman University of Medical Sciences, Kerman, Iran; 4 Department of Nutrition, School of Public Health, International Campus, Iran University of Medical Sciences, Tehran, Iran; 5 Food Security Research Center, Department of Community Nutrition, School of Nutrition and Food Science, Isfahan, Iran; 6 Division of Food Safety and Hygiene, Department of Environmental Health Engineering, School of Public Health, Tehran University of Medical Sciences, Tehran, Iran; 7 Nutrition and Metabolic Diseases Research Center, Ahvaz Jundishapur University of Medical Sciences, Ahvaz, Iran; 8 Student Research Committee, School of Nursing and Midwifery, Guilan University of Medical Sciences, Rasht, Iran; 9 Student Research Committee, Department of Nutrition, Faculty of Medicine, Urmia University of Medical Sciences, Urmia, Iran; 10 School of Paramedicine, Guilan University of Medical Sciences, Langroud, Iran; 11 Cancer Research Center, Shahid Beheshti University of Medical Sciences, Tehran, Iran; 12 Cellular and Molecular Research Center, Sabzevar University of Medical Sciences, Sabzevar, Iran; 13 Faculty of Medicine, Guilan University of Medical Science, Rasht, Iran; 14 Department of Community Nutrition, Faculty of Nutrition and Food Technology, National Nutrition and Food Technology Research Institute, Shahid Beheshti University of Medical Sciences, Tehran, Iran; 15 Non-Communicable Diseases Research Center, Department of Nutrition & Biochemistry, Faculty of Medicine, Sabzevar University of Medical Sciences, Sabzevar, Iran; 16 Department of Clinical Nutrition, School of Nutritional Sciences and Dietetics, Tehran university of medical sciences, Tehran, Iran

**Keywords:** Egg Consumption, Cardiovascular Disease, Stroke, Hypertension, Cardiac ischemia, Myocardial Infarction

## Abstract

**Background:**

Cardiovascular diseases (CVDs) are one of the main causes of death worldwide. Data on the effect of eggs consumption on the risk of CVDs are still unreliable. Therefore, this study aimed to investigate the association between CVDs and the consumption of eggs.

**Methods:**

In this case-control study, the required data were extracted from the Prospective Epidemiologic Research Studies in Iran (PERSIAN) cohort in Sabzevar, Iran. A total of 4241 adults participated including 1535 patients with CVDs as the case group and 2706 healthy people as controls. Egg consumption was assessed using a valid food frequency questionnaire (FFQ).

**Results:**

A significant association was observed between eggs consumption and stroke after adjustment for physical activity, body mass index (BMI), smoking, systolic blood pressure (SBP), diastolic blood pressure (DBP), using alcohol, lipid profile, diabetes, and the intake of energy, lipid, protein, carbohydrate, and cholestrol (OR:1.007, 95% CI:1.001–1.013, *P* = 0.03). No association was found between egg consumption with hypertension, cardiac ischemia, and myocardial infarction.

**Conclusions:**

There was a significant association between the consumption of eggs and stroke. However, more studies are needed to examine the effect of eggs on CVDs.

## Background

Cardiovascular diseases (CVDs), including coronary heart disease (CHD), coronary heart failure, stroke, arterial disease, and high blood pressure (BP), are the leading cause of death worldwide [1, 2]. According to WHO, 31% of deaths worldwide and 20% of deaths in Iran are caused by CVDs [3]. Heart disorders are important health and medical challenges throughout the Eastern Mediterranean region, including Iran [4]. CHD was the leading cause of CVDs-related death (42.6%) in the USA in 2017, followed by stroke (17.0%), hypertension (10.5%), heart failure (9.4%), and artery diseases (2.9%), while all other minor CVDs causes together accounted for 17.6% of deaths [5].

Main CVDs risk factors are aging, male gender, postmenopausal status in women, excess adiposity or obesity, low physical activity, dyslipidemia (e.g., high triglyceride (TG) and/or high low-density lipoprotein cholesterol (LDL-C) levels and/or low high-density lipoprotein cholesterol (HDL-C) levels), diabetes, hypertension, genetic susceptibility, unhealthy diet, and smoking [6, 7]. Regarding dietary intake, the risk factors included low consumption of fruit, vegetable, and fish/fish oil, and high intake of saturated fat, refined carbohydrate, and sodium [6, 7]. Nutritional risk factors have been supposed to be one of the most importance steraregies to reduce the burden of CVDs worldwide [8]. Among many factors widely studied over the last decades, dietary cholesterol is frequently reported to have a strong relationship with increased risk of CVDs [9]. However, recent studies reported contradictory results on the effects of dietary cholesterol on CVDs [10].

One of the main dietary cholesterol sources in the human diet is egg [11, 12]. One egg provides about 5 g of fat, 187 mg of cholesterol, 147 mg of choline, and 6 g of high-quality protein, in addition to iron, vitamins, minerals, and carotenoids according to the USDA [12]. The American Heart Association (AHA) Dietary Guidelines Revision 2000 has advised the public to consume less than 300 mg/day of cholesterol to minimize the elevation of blood cholesterol, despite the lack of concrete proof that eating eggs might cause elevated cholesterol levels [13]. Interestingly, the most recently Dietary Guidelines for Americans 2015–2020 no longer set restrictions on egg consumption, but instead advocate egg consumption as part of a healthy diet [14].

Due to the high cholesterol content of eggs and their possible impact on cardio-metabolic outcomes, there has generally been debate about their effects on human health [15]. A high dietary cholesterol intake was linked to higher blood cholesterol, according to a meta-analysis of 55 studies [16]. A Chinese study also reported that high dietary cholesterol intake was associated with hypercholesterolemia [17]. Also, the increased incidence of stroke with higher egg consumption among type 2 diabetes (T2D) patients was reported in the other studies [18].

On the other hand, some recent systematic reviews and meta-analyses of prospective cohort studies did not find a relationship between egg intake and risk of total CVDs. For example, a recent cohrt study reported that higher egg consumption is not associated with increased risk of CVDs among Iranians. Furthermore, some studies revealed an inverse relationship between egg consumption and risk of CVDs and stroke [19, 20]. Since the prior research on egg consumption and CVD risk has been equivocal and the available information was contentious and inconsistent, we aimed to bridge the knowledge gap by exploring the relationship between egg consumption and different types of CVDs.

## Materials and methods

### Data source

In this case-control study, the required data was extracted from the Prospective Epidemiologic Research Studies in Iran (PERSIAN) in Sabzevar, Iran. The Persian Cohort study Protocol was published elsewhere [21]. A total of 5,000 residents of Sabzevar city, Iran were initially selected by convenient sampling. The inclusion criteria were people with Iranian nationality, households located within the study area, aged 35 to 70 years, consenting to the participation in the study, and recent diagnosis of heart diseases, ischemia, infarction, and high blood pressure for the case group. On the other hand, People who did not want to continue the participation in the study, people who had communication problems and were unable to answer the study questions, those with mental illnesses (characterized by a clinically significant disturbance in an individual’s cognition, emotional regulation, or behaviour [22]), mental retardation, hearing and intellectual impairments or visual loss, and any other disorder that could interfere with the research process were all excluded from both groups. Other exclusion criteria included incomplete medical records, the diagnosis of some serious underlying diseases like cancer, diagnosis of heart diseases more than two months before entering the study, and the use of steroid supplements, barbiturates, and carbamazepine. Finally, 759 people were excluded and a total of 4241 people participated in the study. The case group included 1535 patients (977 patients with hypertension, 435 patients with myocardial ischemia, 70 patients with myocardial infarction, and 53 patients with stroke) and the control group included 2706 healthy adults.

### Data collection

Data were initially collected using valid and reliable questionnaires designed to gather information from participants in PERSIAN cohort, with the approval of the pertinent officials at the PERSIAN Cohort Center in Sabzevar, Iran. The participants were invited to the cohort site and the required data was collected by trained personnels. The collected information was as bellow:

#### Sociodemographic status

A valid and reliable PERSIAN cohort general questionnaire [21] was used to collect information on demographic characteristics, physical activity level, occupation (yes/no), smoking (yes/no), and drinking alcohol (yes/no), medical history (e.g. use of certain specific medications such as statins, heart diseases, diabetes, and hypertension), and family history of heart attack, stroke, and diabetes. Physical activity was measured in four levels: Level 1 activities defined as sedentary work mostly done while sitting (e.g., driving); Level 2 activities defined as standing or occasional walking (e.g., teaching); Level 3 activities defined as mainly indoor activities causing a mild increase in heart rate and sweating (e.g., housekeeping); and Level 4 activities defined as those causing a significant increase in heart rate and sweating usually performed outdoors (e.g., farming). The level of physical activity was represented as the metabolic equivalent of task (MET).

#### Case ascertainment

Three times measurements of systolic (SBP) and diastolic (DBP) blood pressure were performed with a validated sphygmomanometer (Omron M10-IT model, Omron Healthcare, Kyoto, Japan), and the average of the last two measurements was considered for each participant. The measurements were made on the participant’s dominant arm in a seated position after at least 5 min of rest with a cuff of appropriate size, as determined by measurement of the upper arm circumference and following the recommendations of the European Society of Hypertension [23].

The presence of at least two of the following criteria was used to diagnose acute MI: (1) typical chest pain lasting more than 30 min, (2) ST elevation > 0.1 mV in at least two adjacent electrocardiograph leads and (3) an increase in the serum level of cardiac markers (including creatine kinase (CK), creatine kinase-myoglobin binding (CK-MB), CK-MB mass (CK-MBm),

or troponin (cTn) [24]. Stroke was defined based on the definition proposed by the World Health Organization. Accordingly, a rapid-onset focal neurological disorder persisting at least 24 h with probable vascular origin was defined as a stroke case. Ischemic heart disease (IHD) included unstable angina, MI, and sudden cardiac death [25].

#### Anthropometric measurements

Participants’ weight and height were measured with light clothes and without shoes. Weight was measured in kilograms to the nearest 0.1 kg using a mechanical column scale (Seca 755, Germany) and height was measured in centimeters (cm) to the nearest 0.1 cm using a stadiometer (Seca 204, Germany). The BMI of participants was calculated as weight (in kilograms) divided by the square of height (in meters). According to the World Health Organization (WHO), individuals with BMIs of lower than 18.5, 18.5 to 24.9, 25 to 29.9, and 30 kg/m2 and higher were classified as underweight, normal weight, overweight, and obese, respectively [26].

#### Dietary and egg consumption assessment

The data of participant’s habitual food intake was obtained through a reliable and valid semi-quantitative food frequency questionnaire (FFQ), comprising 168 food items usually consumed by Iranians [27]. The frequency of food consumption over the past year was examined through a face-to-face interview. Household measures were taken into account for portion sizes and then were converted to grams. The food composition table (FCT) of the United States Department of Agriculture (USDA) was used to evaluate the amount of energy and nutrients. The Iranian FCT was considered for local foods that were not existed in the FCT. Data collected from FFQ were converted to grams of nutrients using Nutritionist IV software (First Databank Division, the Hearst Corporation, modified for Iranian foods). Based on FFQ, participants were asked about their frequency of habitual egg consumption during the last 12 months.

### Statistical analysis

Descriptive statistics, such as standard deviation, median, and mean for quantitative variables, and number and percentage for qualitative variables were used to describe the subject’s sociodemographic and anthropometric markers. Chi-squared test and independent t-test methods were used for qualitative and quantitative data, respectively. Multivarible loggestic regression was used to assess the association between egg consumption and CVDs including HTN, CI, MI, Stroke, and all CVDs after adjusting the potential confounding factors which were selected based on the results of previous studies. The odds ratio (OR) with a 95% confidence interval (CI) was presented for the occurrence of different types of CVDs. Data analysis was accomplished in SPSS software version 21 (SPSS Inc, Chicago, USA) and a probability level of P < 0.05 was regarded as statistically significant for all analyses.

## Results

The normal distribution of data was analyzed and confirmed using the Kolmogorov-Smirnov statistical test. Table 1 presents data on the general characteristics among the cases and controls. The cases had a higher age (54.19 ± 8.03 vs. 47.20 ± 8.23 y, *P* < 0.001), weight (79.98 ± 13.52 vs. 73.34 ± 13.35 kg, *P* < 0.001), BMI (29.38 ± 4.77 vs. 27.70 ± 4.62 kg/m2, *P* < 0.001), systolic blood pressure (124.59 ± 19.13 vs. 110.36 ± 14.16 mm Hg, *P* < 0.001), diastolic blood pressure (77.40 ± 11.39 vs. 69.73 ± 9.29 mmHg, *P* < 0.001), triglyceride (157.04 ± 92.84 vs. 142.09 ± 105.71 mg/dl, *P* < 0.001), diabetes (26.7 vs. 8.9, p < 0.001) than the controls. On the other hand, the controls had a higher physical activity (39.8 ± 7.99 vs. 37.55 ± 7.17 kcal/kg/d, *P* < 0.001), height (162.76 ± 9.19 vs. 160.84 ± 9.14. cm, *P* < 0.001), serum cholesterol (192.54 ± 38.88 vs.189.65 ± 43.26 mg/dl, *P* = 0.044), low-density lipoprotein (111.92 ± 32.72 vs. 106.41 ± 35.69 mg/dl, *P* < 0.001), occupation (47.1% vs. 30.5%, *P* < 0.001), and smoking (51.6% vs. 36.7%, *P* < 0.001) than the cases.

**Table 1:** The general characteristics of the participants

	Cases (1535)	Controls (2706)	P*
Age (y)	54.19 ± 8.03	47.20 ± 8.23	< 0.001
Height (cm)	160.84 ± 9.14	162.76 ± 9.19	< 0.001
Weight (kg)	75.98 ± 13.52	73.34 ± 13.35	< 0.001
BMI (kg/m2)	29.38 ± 4.77	27.70 ± 4.62	< 0.001
physical activity (kcal/kg/d)	37.55 ± 7.17	39.08 ± 7.99	< 0.001
Right SBP (mm Hg)	124.59 ± 19.13	110.36 ± 14.16	< 0.001
Right DBP (mm Hg)	77.40 ± 11.39	69.73 ± 9.29	< 0.001
TG (mg/dl)	157.04 ± 92.84	142.09 ± 105.71	< 0.001
CHOL (mg/dl)	189.65 ± 43.26	192.54 ± 38.88	0.044
HDL-C (mg/dl)	52.11 ± 10.84	52.50 ± 10.56	0.283
LDL-C (mg/dl)	106.41 ± 35.69	111.92 ± 32.72	< 0.001
Drink alcohol (yes) (n, %)	100(6.5%)	195(7.2%)	0.463
Has Diabetes (yes) (n, %)	409(26.7%)	241(8.9%)	< 0.001
Has Job (yes) (n, %)	468(30.5%)	1274(47.1%)	< 0.001
Smoking (yes) (n, %)	563(36.7%)	1396(51.6%)	< 0.001
Males (n, %)	626(40.8%)	1247(46.1%)	< 0.002
Females (n, %)	909(59.2%)	1458(53.9%)	< 0.002

*Chi-squared test and independent t-test methods were used for qualitative and quantitative data, respectively. Abbreviations: BMI, body mass index; SBP, systolic blood pressure; DBP, diastolic blood pressure; TG, triglyceride; CHOL, cholesterol; HDL-C, high density lipoprotein cholesterol; LDL-C, low-density lipoprotein

Table 2 presents the intake of various nutrients among the participants. The results identified that the cases had a lower intake of protein (74.8.1 ± 25.44 vs. 80.24 ± 25.07 g/d, *P* < 0.001), total fat (61.54 ± 25.68 vs. 65.72 ± 138.31 g/d, *P* < 0.001), carbohydrate (397.46 ± 136.34 vs. 418.99 ± 783.54 g/d, *P* < 0.001), energy (2392.61 ± 779.02 vs. 2541.04 ± 14.9 kcal/d, *P* < 0.001), and cholesterol (237.25 ± 110.16 vs. 268.88 ± 0.26 mg/d, P = 0.002) than the controls. On the other hand, the controls had a lower consumption of egg (25.31 ± 31.73 vs. 74.8 ± 31.73 g/d, *P* < 0.001) than the cases.

**Table 2:** Dietary intake of the participants

	Cases (1535)	Controls (2706)	P*
Protein (g/d)	74.8 ± 25.44	80.24 ± 25.07	< 0.001
Total fat (g/d)	61.54 ± 25.68	65.72 ± 138.31	< 0.001
Carbohydrate (g/d)	397.46 ± 136.34	418.99 ± 783.54	< 0.001
Energy (kcal/d)	2392.61 ± 779.02	2541.04 ± 14.9	< 0.001
Caffeine (mg/d)	195.45 ± 127.82	189.89 ± 64.13	0.202
Total fiber (g/d)	27.23 ± 10.33	27.45 ± 328.02	0.529
Cholesterol (mg/d)	237.25 ± 110.16	268.88 ± 0.26	0.002
Egg (g/d)	74.8 ± 31.73	25.31 ± 31.73	< 0.001

* Independent t-test Abbreviations: MUFA, monounsaturated fatty acid; PUFA, polyunsaturated fatty acid; BCAAs, branched-chain amino acids

The results of logistic regression found a significant association between egg consumption and stroke after adjustment for the confounders (OR:1.007, 95% CI:1.001–1.013, *P* = 0.03) (Table 3). No significant association was found association between egg consumption and the risk of HTN, CI, MI, and all CVDs after adjustment for age and gender (Model 1), further adjustment for physical activity, BMI, SBP, DBP, smoking, drinking alcohol, TG, CHOL, HDL-C, LDL-C, and diabetes (Model 2) and additional adjustment for the intake of calorie, fat, protein, carbohydrate, and cholestrol (Model 3).

**Table 3:** The association of cardiovascular diseases with egg consumption*

	HTN			CI			MI			Stroke			All CVDs	
	OR(CI95%)	P		OR(CI95%)	P		OR(CI95%)	P		OR(CI95%)	P		OR(CI95%)	P
Model 1	1.002 (0.999–1.004)	0.21		1.000 (0.997–1.003)	0.99		1.004 (0.998–1.010)	0.21		1.006 (1.000-1.012)	0.051		1.001 (0.999–1.004)	0.26
Model 2	0.999 (0.996-1.002)	0.39		0.999 (0.996–1.002)	0.55		1.002 (0.995–1.009)	0.53		1.006 (0.999–1.012)	0.09		0.999 (0.996–1.001)	0.42
Model 3	0.999 (0.996-1.001)	0.34		0.999 (0.996–1.003)	0.58		1.002 (0.995–1.009)	0.50		1.007 (1.001–1.013)	0.03		0.999 (0.996–1.001)	0.37

*Loggestic regression Model 1: Adjusted for age and gender, Model 2: Further adjusted for physical activity, BMI, SBP, DBP, smoking, using alcohol, TG, CHOL, HDL-C, LDL-C, and diabetes, Model 3: Further adjusted for energy, lipid, protein, carbohydrate, and cholestrol

Abbreviations: HTN, hypertension; CI, cardiac ischemia; MI, myocardial infarction; CVDs, cardiovascular diseases

## Discussion

**Fig. 1:**
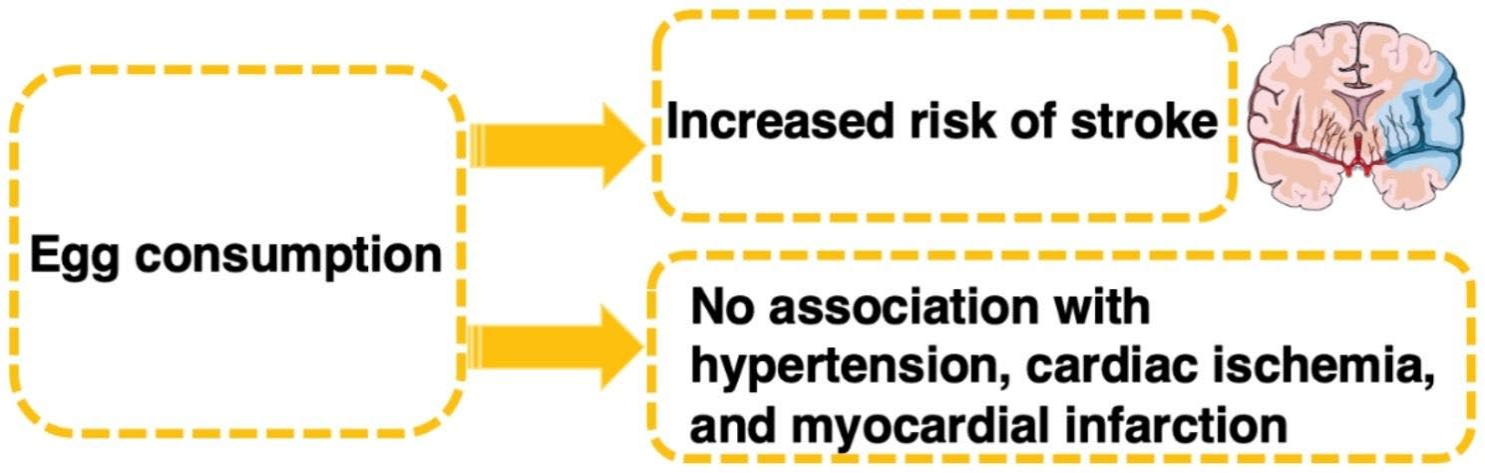
Egg consumption and risk of cardiovascular disease

The present study investigated the association between egg consumption and the risk of cardiovascular diseases. The results showed that the cases had a higher egg consumption than the controls. There was a positive association between dietary intake of egg and stroke after adjustment for physical activity, BMI, SBP, DBP, smoking, drink alcohol, TG, CHOL, HDL, LDL-C, diabetes, and the intake of calorie, lipid, protein, carbohydrate, and cholesterol (Fig. 1). No significant association was observed between egg consumption and the risk of HTN, CI, MI, and All CVDs.

Some studies reported that higher consumption of eggs increased the chance of developing CVDs and high level of dietary cholesterol was reported to be responsible for this negative effect [28]. However, some other studies did not find a relationship between egg consumption and the risk of CVDs or revealed an inverse relationship between egg consumption and the risk of CVDs [20, 29]. For example, Drouin-Chartier et al. assessed the result of 3 cohorts and indicated that egg consumption is not related to heart diseases and on the contrary, it may decrease the risk of CVDs in Asian populations [20]. In addition, a meta-analysis identified that intake of more than 1 egg per day decreases the risk of coronary artery disease [30]. The differences in the obtained results may be due to the influence of other dietary components on the risk of CVDs.

Moreover, the association between CVDs and the consumption of egg may be influenced by the amount of egg consumption. A cohort study by Godos et al. found that a moderate intake of eggs (4/week) did not increase CVDs risk while a high intake of eggs (1/day) is related to heart failure [31]. Another cohort study which was done among Chinese adults indicated that a moderate level of egg intake (5–6/week) was associated with a reduced risk of CVDs including a 26% lower risk of hemorrhagic stroke and a 12% lower risk of IHD [32]. A positive correlation between CVDs and eggs was found among people who consumed more than 250 mg/day of cholesterol [33]. Intake of ≥ 5 eggs per week was related to a 10% increased risk of CVDs, especially among diabetes and obese patients [34]. In another study of six cohorts, the findings showed an 8% increased risk of coronary heart disease with each additional consumption of half an egg [28]. However, in the present study, egg consumption was considered as a continius varible and higher consumption of eggs was only correlated with higher risk of stroke. Another reason for the contradictory results between studies may depend on the variation in response to dietary cholesterol among subjects. Although most people experience moderate to no change in blood cholesterol following dietary cholesterol intake (described as “normal responders”), about a third of people develop an abnormal increase in circulating LDL-C as a result of an increased fractional absorption and/or endogenous synthesis of cholesterol in response to the intake of dietary cholesterol (described as “hyper responders”) [35]. An unusual response to dietary cholesterol has been hypothesized that is depended to altered cholesterol transport due to increased levels of apolipoprotein C-III and decreased apolipoprotein E [36].

The mechanisms that eggs can influence the risk of CVDs is not clear. Eggs are one of the dietary rich sources of cholesterol whichl is considered a risk factor for CVDs [28]. Moreover, egg consumption has been reported to be correlated with other unhealthy behaviors such as smoking, unhealthy dietary patterns, and low physical activity [37]. Also, cholesterol-rich foods such as eggs are usually high in animal proteins and saturated fats [38]. A reduction of dietary cholesterol intake, in addition to the isocaloric replacement of saturated fat by unsaturated fat, was significantly associated with reduced serum cholesterol level [39]. Interestingly, the results of the present study indicated that dietary intake of cholesterol was lower in the case group and the positive association between the consumption of egg and stroke appeared to be significant even after adjustment for cholestrol. So, it is possible that the adverse association of eggs and stroke is independent from the cholesterol content of eggs. The increased risk of heart disease may be related to the choline contents of the eggs. Part of the choline may convert to trimethylamine by gut microbiota, which in turn is oxidized in the liver to trimethylamine-N-oxide (TMAO), which is associated with increased atherosclerosis [40].

However, this study has some limitations. First, appropriate interpretation of the study findings requires consideration of measurement bias for self-reported dietary data. Second, generalizing the results to non-Iranian populations requires caution due to different nutritional habits and different chronic disease epidemiology. Third, this study was observational and cannot establish causality. Fourth, the FFQ that was used for assessing dietary intake does not give information about the egg preparation methods (boiled, scrambled, and fried eggs) which may influence the contents of eggs. Despite some limitations, our study had some strengths. Large sample size, sub analyze for different types of CVDs, and adjusting for different confounders in different models were some advantages of this study. According to the findings of the study, there was no significant association between the consumption of eggs and the risk of CVDs, except for stroke. Further studies should be performed to establish a causal relationship between eggs consumption and the risk of CVDs.

## Conclusion

There was a significant association between egg intake and stroke. I be confirmed in future studies, dietary recommendations for egg consumption should be adjusted for people at risk of stroke. Further longitudinal studies are warranted to investigate the effect of egg consumption CVDs and to discover the underlying mechanisms.

## Data Availability

The datasets used and/or analysed during the current study available from the corresponding author on reasonable request.
